# STeroids Against Radiculopathy (STAR) trial: a statistical analysis plan

**DOI:** 10.1186/s13063-020-05018-2

**Published:** 2021-01-22

**Authors:** Bastiaan C. ter Meulen, Johanna M. van Dongen, Marinus van der Vegt, Henry C. Weinstein, Raymond W. J. G. Ostelo

**Affiliations:** 1 Department of Neurology, OLVG Amsterdam, Jan Tooropstraat 164, 1061 AE Amsterdam, The Netherlands; 2 Department of Epidemiology and Biostatistics Amsterdam Movement Sciences Research Institute, Amsterdam UMC, Vrije Universiteit Amsterdam, De Boelenlaan 1089a, 1081 HV Amsterdam, The Netherlands; 3 Department of Health Sciences, Faculty of Science, Vrije Universiteit Amsterdam, Amsterdam Movement Sciences, de Boelelaan 1085, 1081 HV Amsterdam, The Netherlands; 4 Department of Anesthesiology Zaans Medisch Centrum, Zaandam, The Netherlands

**Keywords:** Sciatica, Lumbar disc herniation, Transforaminal epidural steroids, Economic evaluation, Randomized controlled trial

## Abstract

**Background:**

Transforaminal epidural injections with steroids (TESI) are used increasingly for patients with sciatica. However, their safety, effectiveness, and cost-effectiveness are still a matter of debate. This a priori statistical analysis plan describes the methodology of the analysis for the STAR trial that assesses the (cost-)effectiveness of TESI during the acute stage of sciatica (< 8 weeks).

**Methods:**

The STAR trial is a multicentre, randomized controlled, prospective trial (RCT) investigating the (cost-)effectiveness of TESI by making a three-group comparison among patients with acute sciatica due to a herniated lumbar disc (< 8 weeks): (1) TESI combined with levobupivacaine added to oral pain medication (intervention group 1) versus oral pain medication alone (control group), (2) intervention group 1 versus transforaminal epidural injection with levobupivacaine and saline solution added to oral pain medication (intervention group 2), and (3) intervention group 2 versus control group. Co-primary outcomes were physical functioning (Roland Morris Disability Questionnaire), pain intensity (10-point numerical rating scale), and global perceived recovery (7-point Likert scale, dichotomized into ‘recovered’ and ‘not recovered’). For all three comparisons, we defined the following minimal clinically relevant between-group differences: two points for pain intensity (range 0–10), four points for physical functioning (range 0–24) and a 20% difference in recovery rate. Secondary outcomes are health-related quality of life (EQ-5D-5L) and patient satisfaction (7-point Likert scale) and surgery rate. We also collected resource use data to perform an economic evaluation. Analyses will be conducted by intention-to-treat with *p* < 0.05 (two-tailed) for all three comparisons. Effects will be estimated using mixed models by maximum likelihood. For each comparison, mean differences, or difference in proportions, between groups will be tested per time point and an overall mean difference, or difference in proportions, between groups during the complete duration of follow-up (6 months) will be estimated. In the economic evaluation, Multivariate Imputation by Chained Equations will be used to handle missing data. Cost and effect differences will be estimated using seemingly unrelated regression, and uncertainty will be estimated using bootstrapping techniques.

**Discussion:**

This statistical analysis plan provides detailed information on the intended analysis of the STAR trial, which aims to deliver evidence about the (cost-)effectiveness of TESI during the acute phase of sciatica (< 8 weeks).

**Trial registration:**

Dutch National trial register NTR4457 (6 March 2014)

**Supplementary Information:**

The online version contains supplementary material available at 10.1186/s13063-020-05018-2.

## Update

Sciatica or lumbar radicular syndrome is a disabling condition characterized by radiating leg pain, with or without low back pain [1]. Sciatica may be accompanied by neurological deficits, such as weakness of the leg muscles or sensory loss. About 85% of sciatica cases are caused by lumbar disc herniation [2]. During the first few weeks after onset, treatment primarily focusses on pain reduction and improvement of physical functioning (https://www.nice.org.uk/guidance/ng59/chapter/Recommendations). Pain medication and physiotherapy are usually initiated by the general practitioner. If patients are referred to a hospital in case of moderate to severe pain or neurological deficits, they are typically treated with epidural corticosteroid injections or surgery. However, the effectiveness, cost-effectiveness and safety [3–6] of epidural corticosteroid injections are still a matter of debate and therefore more high-quality RCTs are needed.

The STAR trial (STeroids Against Radiculopathy) assesses both the effectiveness and cost-effectiveness of transforaminal epidural injections with steroids (TESIs) in patients with acute sciatica (< 8 weeks post onset). This is done by making the following three comparisons: (1) TESI combined with levobupivacaine added to oral pain medication (intervention group 1) versus oral pain medication alone (control group), (2) intervention group 1 versus transforaminal epidural injection with levobupivacaine and saline solution added to oral pain medication (intervention group 2), and (3) intervention group 2 versus control group. Our hypothesis is that intervention-1 group will experience less pain and better physical functioning compared to both the control group and intervention group 2 and that intervention group 2 is more effective than the control group as well. Hence, these interventions will be assessed for superiority.

Participant recruitment commenced in January 2016 and was completed in November 2019. Data collection was completed in April 2020. This statistical analysis plan details the planned analyses for the STAR trial to facilitate transparency of our data analyses and was developed according to appropriate guidelines [7]. The initial statistical analysis plan was approved and signed by the study investigators on April 23th 2020 and was revised on September 1, 2020. All of the statistical analyses will be performed following data integrity checks and locking and will be commenced in October 2020.

## Study overview

### Trial design

The STAR trial is a multicentre, randomized controlled, prospective trial that investigates the effectiveness and cost-effectiveness of TESI by making a three-group comparison among patients with acute sciatica due to a herniated lumbar disc (< 8 weeks): (1) TESI combined with levobupivacaine added to oral pain medication (intervention group 1) versus oral pain medication alone (control group), (2) intervention group 1 versus transforaminal epidural injection with levobupivacaine and saline solution added to oral pain medication (intervention group 2), and (3) intervention group 2 versus control group. Follow-up is 6 months. On March 6, 2014, the protocol was registered at the Dutch Trial Register (number NTR 4457). On August 20, 2015, the design of the STAR trial was approved by the Medical research Ethics Committees United, Nieuwegein, The Netherlands (registration number NL 45805.100.15) and the study protocol has been published elsewhere [8].

### Study population

Between January 13, 2016, and September 10, 2019, 141 eligible participants [8], who were seeking care for their back-related leg pain (sciatica), were recruited from two Dutch Neurology outpatient clinics (i.e. the Zaans Medisch Centrum, Zaandam and OLVG, Amsterdam, The Netherlands).

To be eligible for this study had to have < 8 weeks of sciatic symptoms and had to be seen by a neurologist in one of the two study centres upon referral by their general practitioners (GP). Additional inclusion criteria were (a) age between 18 and 75 years, (b) a magnetic resonance imaging (MRI) confirmed disc herniation with nerve root impingement causing clinical symptoms, (c) an average pain intensity of > 4 on a 10-point numerical rating scale (NRS) during the last week, (d) good understanding of the Dutch language, and (e) Internet access in order to be able to complete online questionnaires [8]. Exclusion criteria were (a) severe weakness of the legs (Medical Research Council [MRC] score < 3), (b) spinal surgery during the previous year at the symptomatic lumbar level, (c) lumbar spinal stenosis or spondylolisthesis as the cause of radicular pain diagnosed by MRI, (d) pregnancy, and (e) severe comorbidity (e.g. cancer) [8].

### Sample size

We had initially aimed to include 264 patients (*n* = 88 per arm) [8]. This sample size was based on the three co-primary outcomes (i.e. pain, physical functioning, and global perceived effect), a 10% loss to follow-up, a power of 0.9, and a two-sided alpha of 0.05. We calculated that 48 patients would be needed per arm to detect a minimal clinical important difference of 20 points (SD = 30) for both leg and back pain on a 10-point NRS between intervention group 1 and control [9]. Moreover, 22 patients were estimated to be required per arm to detect a difference of 4 points (SD = 4) on the RDMQ-24 scale and 79 patients per arm to detect a difference on the dichotomized GPE of 20%. Unfortunately, this sample size was not reached, as the trial was stopped prematurely due to slow participant accrual. Stopping the trial was a decision by the research team only, meaning that there was no data monitoring board involved, and was based on prior evidence that very few trials with less than 50% of the required sample size at 1 to 2 years after launch ultimately attain sufficient accrual [10]. When trial inclusion stopped at September 10, 2019, 46, 50, and 45 patients were randomized to intervention group 1, intervention group 2, and control, respectively. Consequently, the analyses will likely to be slightly underpowered for pain intensity and global perceived effect, but not for physical functioning.

### Randomization and treatment allocation

After providing informed consent and completing baseline questionnaires, eligible patients were randomized, stratified for treatment centre, by the study coordinator (BTM) using ALEA® software (NKI-AVL, The Netherlands). Alea® generated a random schedule of blocks with a maximum size of 6. Allocation across groups was at a 1:1:1 ratio.

### Study conditions

A detailed description of the study conditions can be found in the design article [8].

In brief, the transforaminal epidural injection procedure was similar for intervention group 1 and intervention group 2. That is, the study participant was brought to a fluoroscopy room and placed in a prone position on the procedure table. Fluoroscopy was used for localization of MRI confirmed disc herniation. Target identification and needle entry into the targeted space was done following internationally accepted procedures [9]. In short, the skin was made sterile using chlorhexidine. The injections were given with 22 gauge 100 mm facet tipped needle (Pajunk RGN™). Right needle position was confirmed with the injection of 0.5–1.5 cc of Joversol 300 mg/ml contrast material (Optiray™ 300, Mallinckrodt). Once an image was obtained demonstrating contrast material spreading into the epidural space medial to a line connecting the ipsilateral lumbar vertebral pedicles, the injection was performed [8].

Patients in intervention group 1 received 1 ml of 5% levobupivacaine followed by 1 ml of 40 mg/ml methylprednisolone in an opaque syringe. Patients in intervention group 2 received 1 ml 5% levobupivacaine followed by 1 ml NaCl 0.9% [8].

All treatment groups were allowed to use oral pain medication and were permitted to go to a physiotherapist in case of kinesiophobia and/or an inactive lifestyle. All oral pain medication during the trial was registered by the participants themselves in Survalyzer, an online questionnaire (www.survalyzer.com). All patients participating in the trial underwent MR Imaging of the lumbar spine that was evaluated by a radiologist (see design article for scan protocol) [8].

### Protocol deviations

Protocol deviations were defined as intervention group 1 and intervention group 2 patients who received no epidural injection, more epidural injections than prescribed by the study protocol, and/or a type of injection fluid other than the one prescribed by the study protocol. For the control group, protocol deviations were defined as patients who received one or more epidural injections in spite of being randomized to the oral pain medication alone condition. Protocol deviations will be confirmed prior to database lock for the final analysis. All protocol violators will be included in the main analysis and a per-protocol analysis will be performed to assess the impact of protocol deviations if more than 10% of the patients will be found to have deviated from the protocol.

### Blinding

This pragmatic trial was partially blinded. Patients in intervention group 1 and intervention group 2 did not know which type of injection they received. However, the type of injection fluid was known to the anaesthesiologist performing the injections. Neurologists who performing the clinical follow-up of the patients were blinded for the type of injection (intervention group 1 versus intervention group 2). The same applied to research nurses.

### Patient characteristics and study outcomes

Patients were asked to complete a web-based questionnaire, containing descriptive questions as well as questions on clinical outcomes and resource use, at baseline, 3 and 6 weeks, and 3 and 6 months after randomization using Survalyzer (www.survalyzer.com). The neurological examination at baseline, length and weight were registered in Openclinica for clinical data (https://www.openclinica.com/). Table 1 gives a schematic overview of the data collection process.

**Table 1:** Overview of the data collection

Outcome measures	Baseline	Follow-up			
		3 weeks	6 weeks	12 weeks	26 weeks
Baseline measurements					
Demographic data	X				
Prognostic factors	X				
Complaint history	X				
Physical examination	X				
MRI lumbar spine	X				
Primary outcomes					
Leg pain intensity (VAS)	X	X	X	X	X
Back pain intensity (VAS)	X	X	X	X	X
Global perceived effect (GPE)		X	X	X	X
Functional status (RDQ)	X	X	X	X	X
Secondary outcomes					
Work status	X	X	X	X	X
Quality of life (EQ-5D-5L)	X	X	X	X	X
Drug use	X	X	X	X	X
Other resource use (cost questionnaire)	X			X	X
Surgery					X

### Baseline measurement

At baseline, all primary and secondary outcomes were measured and additional information was collected on:

- Demographics: age, gender, length and weight, education level, work and marital status.
- Episode details: back and leg pain duration.
- Neurological examination: physical examination of the leg muscles using the Medical Research Council (MRC) scale for muscle strength; sensory examination: tests for perception of light touch, pin prick, and vibration sense of the lower extremities; reflex examination: tests for reflexes in the patellar (L4) and ankle (S1); straight leg raising (or Lasègue’s sign) and a finger-floor distance. Straight leg raising was considered positive if the patient experienced radicular pain when the leg is at an angle < 60°. A finger-floor distance of more than 25 cm was considered indicative for a herniated disc.
- Magnetic resonance imagining: level and side of disc herniation.

### Co-primary outcomes

Our co-primary outcomes included pain intensity (back and leg), physical functioning and global perceived recovery and were assessed at baseline, 3 and 6 weeks, and 3 and 6 months.

Pain intensity was assessed by asking patients about their average pain during the previous week, in both the back and the leg, and was rated using a 10-point NRS: 0 = no pain to 10 = worst imaginable pain [11].

Physical functioning was assessed using the Dutch version of the Roland Morris Disability Questionnaire (RDMQ) [12]. The RDMQ includes 24 items assessing normal daily activities. Each question has a ‘yes’ or ‘no’ option and the overall RDMQ-24 scale ranges from 0 to 24, with higher values indicating more physical limitations [13].

Global perceived recovery (GPR) was rated on a 7-point Likert scale, ranging from ‘completely recovered’ to ‘worse than ever’. The GPR was dichotomized into recovered (categories ‘completely’ and ‘much recovered’) and (categories ‘slightly recovered’, ‘no change’, ‘slightly worse’, ‘much worse’ and ‘worse than ever’) [14].

For the co-primary outcomes, we defined the following minimal clinically relevant between-group differences for all three comparisons: two points for pain intensity (range 0–10), four points for physical functioning (range 0–24) and a 20% difference in recovery rate across groups [9]. In accordance with the guidelines of the ‘European Medicines Agency’, we will only consider one intervention effective over another, if statistically significant and clinically relevant differences are found between them for all co-primary outcomes (https://www.ema.europa.eu/en/documents/scientific-guideline/draft-guideline-multiplicity-issues-clinical-trials_en.pdf) and therefore we will not adjust our analyses for multiplicity induced by having co-primary outcomes. We will also not adjust for multiplicity induced by having 3 comparators, because we will conduct various pairwise comparisons (i.e. intervention 1 versus control, intervention 2 versus control, intervention 1 versus control) with a clear hierarchy in anticipated effectiveness (i.e. intervention 1 > intervention 2 > control), instead of a global test of unordered groups [15].

### Secondary outcomes

Secondary outcomes included health-related quality of life, patient satisfaction and surgery rate and were assessed at baseline, 3 and 6 weeks, and 3 and 6 months.

Health-related quality of life was assessed using the Euroqol-5 dimensions-5 levels (EQ-5D-5L) [16]. The EQ-5D-5L asks patients to rate the severity of their health problems (levels: no problems, slight problems, moderate problems, severe problems, unable to/extreme problems) on five health dimensions (health dimensions: mobility, self-care, usual activities, pain/discomfort, anxiety/depression). The patients’ resulting EQ-5D-5L health states will be converted to utility values ranging from 0 (equal to death) to 1 (equal to full health) using the Dutch tariff [17]. For the economic evaluation, quality-adjusted life years will be estimated by multiplying the time spent in a certain health state by its respective utility value.

Patient satisfaction was assessed using a 7-point Likert scale ranging from ‘not satisfied at all’ to ‘completely satisfied’ [18].

Surgery rate was assessed by keeping track of whether or not patients needed surgery in spite of conservative treatment (control group) and a possible epidural injection recorded in the case record form (CRF). Surgery rate was measured as dichotomous outcome, indicating whether patients received a surgery during follow-up (yes = 1/no = 0).

### Confounding factors

Confounding factors were a priori selected based on evidence from existing studies in sciatica, and expertise within the study team [19, 20]. The factors were age, gender, body mass index (BMI) and severity of back and leg pain at baseline.

### Societal costs

Intervention costs will be estimated using a micro-costing approach. That is, detailed information on the number of TESIs performed per patient and the cost per TESI were collected from hospital accounting records. Information regarding the use of all other kinds of resources was collected using online cost questionnaires administered at 3 weeks, 6 weeks, and 3 and 6 months. See supplemental file for the questionnaire. For assessing absenteeism and presenteeism, slightly adapted versions of the World Health Organization – Work Performance Questionnaire (WHO-HPQ) and the iMTA Productivity Cost Questionnaire (iPCQ) were used, respectively [21, 22]. Healthcare utilization, consisting of the use of primary care, secondary care, prescribed and over-the-counter medication, were valued using Dutch standard prices and unit prices derived from http://www.medicijnkosten.nl. If unavailable, prices according to professional organizations were used. Informal care and unpaid productivity losses were valued using a recommended Dutch shadow price [23]. Absenteeism was valued in accordance with the Friction Cost Approach, with a friction period of 12 weeks, and using gender-specific price weights. Presenteeism was valued using gender-specific price weights as well [24]. All costs were converted to Euros 2020 using consumer price indices.

### Adverse events and safety issues

All adverse events (AEs) during the study were recorded on the case record form (CRF), whether or not caused by the study procedure. Registration included: the event, onset and end date, severity, relation to the study and action taken. AEs considered related to the study were judged by a medically qualified investigator and followed until resolution (or if the event was regarded stable). There were no AEs that resulted in withdrawal from the trial.

### Registering and handling of data

A trial master file was established in Amsterdam by the coordinating investigator (BTM). The registering of data was done consecutively throughout the study. Data were registered in a case report form (CRF) for each patient. Throughout the study, the registering and handling of data followed the principles of good clinical practice (GCP). The data will be kept in a locked facility for 15 years after the study is finished. After this, it will be destroyed. The statistical master file is kept by the department of Data Management, at Amsterdam UMC (location VUMC) Amsterdam, the Netherlands.

## Statistical analysis

All analyses described in this plan are considered a priori analyses in that they have been defined in the study protocol and/or this SAP. All post hoc analyses will be identified as such in the article. Analyses will be consistent with the intention-to-treat principle and will be performed using software package SPSS v26 and STATA v16.

### Trial profile

**Fig. 1:**
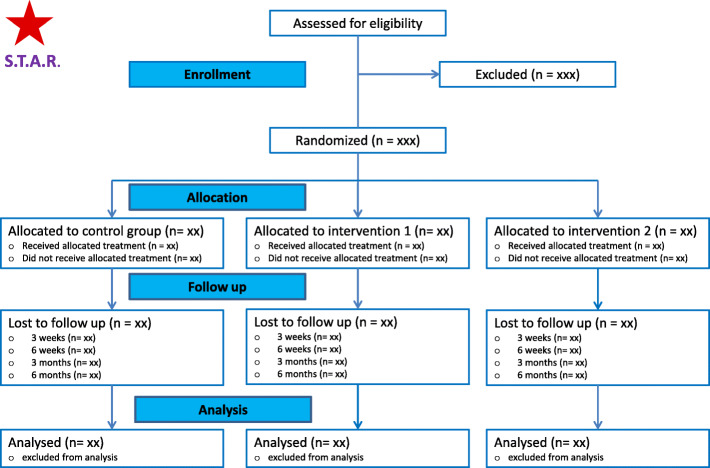
STAR trial: enrollment and randomization

The following summaries will be presented in a flow diagram according to the CONSORT statement [25]: the number of patients with acute sciatica that were screened for eligibility at the Neurology outpatient clinics in Amsterdam and Zaanstad and the number of patients that was eventually randomized after providing informed consent, stratified for each treatment group. Additionally, the number and percentages of patients lost to follow-up will be reported per treatment arm, including information about the timing and reason(s) for loss to follow-up. See Fig. 1 for an overview.

### Data integrity

Prior to the analyses, the integrity of trial data will be checked by scrutinizing data files for omissions and errors. The source of any inconsistencies will be explored and resolved.

### Methods for handling missing data

In the effect analyses, missing data will be handled using mixed models by maximum likelihood estimation [26]. In case more than 10% of patients have missing data, defined as having missing data on one or more variable, sensitivity analyses will be performed using mixed models with multiple imputation. For the economic evaluation, missing data will be multiply imputed, irrespective of the percentage of patients of missing data. This strategy is advised in economic evaluations, because total costs are the sum of numerous cost components, so total costs will already be missing if only one item is missing. Data will be multiply imputed using Multivariate Imputation by Chained Equations (MICE) and the number of imputed datasets will be determined using the loss of efficiency approach [27]. Imputation models will include all available cost and effect measure values as well as variables differing between groups at baseline, those variables related to the ‘missingness’ of data and variables related to the outcomes. Pooled estimates will be calculated using Rubin’s rules.

### Evaluation of demographics and baseline patient characteristics

Demographic baseline characteristics will be described per treatment group (Table 2). Continuous variables will be summarized using standard measures of central tendency and dispersion, as either mean and standard error (data that with a normal distribution) or median and interquartile range (data with a skewed distribution). Dichotomous or categorical variables will be summarized by frequencies and percentages. In accordance with the CONSORT statement, we will not statistically test whether baseline differences across study groups [25, 28].

**Table 2:** Baseline variables

	Control group (*n* = xxx)	Intervention group 1 (*n* = xxx)	Intervention group 2 (*n* = xxx)
**Participants characteristics**			
Female	*n*/*N* (%)	*n*/*N* (%)	*n*/*N* (%)
Age (years)	M ± SD	M ± SD	M ± SD
BMI	M ± SD	M ± SD	M ± SD
Vascular risk factors—no. (%)	x (%)	x (%)	x (%)
Joint problems—no. (%)	x (%)	x (%)	x (%)
Education level—no. (%)^a^			
Low	x (%)	x (%)	x (%)
Moderate	x (%)	x (%)	x (%)
High	x (%)	x (%)	x (%)
Married or with a partner—no. (%)	x (%)	x (%)	x (%)
Having a paid job—no. (%)	x (%)	x (%)	x (%)
**Neurological examination**			
Motor deficit— no. (%)	x (%)	x (%)	x (%)
Sensory deficit—no. (%)	x (%)	x (%)	x (%)
Pain on straight leg raising—no. (%)	x (%)	x (%)	x (%)
**Level of herniation (MRI)- no (%)**			
L3–4	x (%)	x (%)	x (%)
L4–5	x (%)	x (%)	x (%)
L5–S1	x (%)	x (%)	x (%)
**Primary outcomes**			
Leg pain intensity score^b^	M ± SD	M ± SD	M ± SD
Back pain intensity score^b^	M ± SD	M ± SD	M ± SD
Functional status^c^	M ± SD	M ± SD	M ± SD
**Secondary outcomes**			
Quality of life^d^			

*BMI* body mass index (calculated as weight in kilogrammes divided by height in metres squared)

^a^Low indicates preschool, primary school or lower secondary school; moderate indicates higher secondary school or undergraduate; high indicates tertiary, university or postgraduate

^b^Measured by numeric rating scale (score range, 0–10); a higher score indicates more severe pain intensity

^c^Measured by Roland Disability Questionnaire (score range, 0–24); a higher score indicates worse functioning

^d^Measured by EQ-5D-5 L (score range, 0–1); a higher score indicates better quality of life

### Primary analysis

All statistical tests of the primary and secondary analyses will be 2-sided and both 95% confidence intervals (95% CIs) and *p* values will be reported. Moreover, as indicated above, three pairwise comparisons will be conducted per outcome: (1) intervention 1 versus control, (2) intervention 2 versus control, and (3) intervention 1 versus control. The assumption of normal distribution will be checked by visual inspection and using a QQ-plot. Non-normally distributed data will be log-transformed. If normality will not be achieved after log-transformation, data will be dichotomized into either having a minimal clinically important improvement or not (e.g. ≥ 2 points for pain and ≥ 4 points for physical functioning [yes/no]). Pain intensity (back and leg) and physical functioning will be assessed using linear mixed models by maximum likelihood and global perceived recovery will be assessed using logistic mixed models by maximum likelihood. All models will have a 2-level structure, with time clustered within patients. Linear and logistic mixed models will be fitted using an ‘independent’ covariance matrix for the random effects, which allows for a distinct variance for each random effect within a random-effects equation and assumes that all covariances are 0. Linear mixed models will also use a large-sample approximation of the sampling distribution of the test statistic and will assume that all residuals are independent and identically distributed Gaussian with a common variance (www.stata.com). The necessity of having a random intercept and/or a random slope will be assessed using the likelihood-ratio test. For all co-primary outcomes, the main effect will be the pooled mean difference, or difference in proportions, across groups during the complete duration of follow-up. Additionally, mean differences, or differences in proportions, across groups will be tested per time point using time by treatment interactions. For all co-primary outcomes, adjusted (adjusted for the predefined confounders; see above) and unadjusted analyses will be performed and presented (Table 3).

**Table 3:** Primary outcomes according to treatment and timing

		**Intervention group 1, mean (SD)**	**Intervention group 2, mean (SD)**	**Control group, mean (SD)**	**Comparison 1, treatment effect, mean difference (95% CI)**	**Comparison 2, treatment effect, mean difference (95% CI)**	**Comparison 3, treatment effect, mean difference (95% CI)**
**Outcome**							
**Back pain**	**Overall effect**				**X (XX–XX)_**	**X (XX–XX)_**	**X (XX–XX)_**
	Baseline	X (XX)	X (XX)	X (XX)	X (XX–XX)_	X (XX–XX)_	X (XX–XX)_
	3 weeks	X (XX)	X (XX)	X (XX)	X (XX–XX)_	X (XX–XX)_	X (XX–XX)_
	6 weeks	X (XX)	X (XX)	X (XX)	X (XX–XX)_	X (XX–XX)_	X (XX–XX)_
	12 weeks	X (XX)	X (XX)	X (XX)	X (XX–XX)_	X (XX–XX)_	X (XX–XX)_
	26 weeks	X (XX)	X (XX)	X (XX)	X (XX–XX)_	X (XX–XX)_	X (XX–XX)_
**Leg pain**	**Overall effect**				**X (XX–XX)_**	**X (XX–XX)_**	**X (XX–XX)_**
	Baseline	X (XX)	X (XX)	X (XX)	X (XX–XX)_	X (XX–XX)_	X (XX–XX)_
	3 weeks	X (XX)	X (XX)	X (XX)	X (XX–XX)_	X (XX–XX)_	X (XX–XX)_
	6 weeks	X (XX)	X (XX)	X (XX)	X (XX–XX)_	X (XX–XX)_	X (XX–XX)_
	12 weeks	X (XX)	X (XX)	X (XX)	X (XX–XX)_	X (XX–XX)_	X (XX–XX)_
	26 weeks	X (XX)	X (XX)	X (XX)	X (XX–XX)_	X (XX–XX)_	X (XX–XX)_
**Physical functioning**	**Overall effect**				**X (XX–XX)_**	**X (XX–XX)_**	**X (XX–XX)_**
	Baseline	X (XX)	X (XX)	X (XX)	X (XX–XX)_	X (XX–XX)_	X (XX–XX)_
	3 weeks	X (XX)	X (XX)	X (XX)	X (XX–XX)_	X (XX–XX)_	X (XX–XX)_
	6 weeks	X (XX)	X (XX)	X (XX)	X (XX–XX)_	X (XX–XX)_	X (XX–XX)_
	12 weeks	X (XX)	X (XX)	X (XX)	X (XX–XX)_	X (XX–XX)_	X (XX–XX)_
	26 weeks	X (XX)	X (XX)	X (XX)	X (XX–XX)_	X (XX–XX)_	X (XX–XX)_
		**Intervention group,** ***N*** **(%)**		**Control group (%),** ***N*** **(%)**	**Treatment effect, odds ratio (95% CI)**	**Treatment effect, odds ratio (95% CI)**	**Treatment effect, odds ratio (95%CI)**
**Global Perceived Effect**	**Overall effect**				**X (XX–XX)_**	**X (XX–XX)_**	**X (XX–XX)_**
	3 weeks	X (XX)	X (XX)	X (XX)	X (XX–XX)_	X (XX–XX)_	X (XX–XX)_
	6 weeks	X (XX)	X (XX)	X (XX)	X (XX–XX)_	X (XX–XX)_	X (XX–XX)_
	12 weeks	X (XX)	X (XX)	X (XX)	X (XX–XX)_	X (XX–XX)_	X (XX–XX)_
	26 weeks	X (XX)	X (XX)	X (XX)	X (XX–XX)_	X (XX–XX)_	X (XX–XX)_

Note: Comparison 1: intervention group 1 versus control; comparison 2: intervention group 2 versus control; comparison 3: intervention group 1 versus Intervention group 2

### Secondary analysis

Secondary outcomes health-related quality of life and satisfaction will be analysed using the same linear mixed models as the primary analysis. Surgery rate will be assessed using a logistic regression and both an adjusted and an unadjusted analysis will be performed and presented (Table 4).

**Table 4:** Secondary outcomes according to treatment and timing

		Intervention group 1, mean (SD)	Intervention group 2, mean (SD)	Control group, mean (SD)	Comparison 1, treatment effect, mean difference (95% CI)	Comparison 2, treatment effect, mean difference (95% CI)	Comparison 3, treatment effect, mean difference (95% CI)
**Outcome**							
**Health-related quality of life**	**Overall effect**				**X (XX–XX)_**	**X (XX–XX)_**	**X (XX–XX)_**
	Baseline	X (XX)	X (XX)	X (XX)	X (XX–XX)_	X (XX–XX)_	X (XX–XX)_
	3 weeks	X (XX)	X (XX)	X (XX)	X (XX–XX)_	X (XX–XX)_	X (XX–XX)_
	6 weeks	X (XX)	X (XX)	X (XX)	X (XX–XX)_	X (XX–XX)_	X (XX–XX)_
	12 weeks	X (XX)	X (XX)	X (XX)	X (XX–XX)_	X (XX–XX)_	X (XX–XX)_
	26 weeks	X (XX)	X (XX)	X (XX)	X (XX–XX)_	X (XX–XX)_	X (XX–XX)_
**Patient satisfaction**	**Overall effect**				**X (XX–XX)_**	**X (XX–XX)_**	**X (XX–XX)_**
	Baseline	X (XX)	X (XX)	X (XX)	X (XX–XX)_	X (XX–XX)_	X (XX–XX)_
	3 weeks	X (XX)	X (XX)	X (XX)	X (XX–XX)_	X (XX–XX)_	X (XX–XX)_
	6 weeks	X (XX)	X (XX)	X (XX)	X (XX–XX)_	X (XX–XX)_	X (XX–XX)_
	12 weeks	X (XX)	X (XX)	X (XX)	X (XX–XX)_	X (XX–XX)_	X (XX–XX)_
	26 weeks	X (XX)	X (XX)	X (XX)	X (XX–XX)_	X (XX–XX)_	X (XX–XX)_
**Total number of surgeries performed**	26 weeks	X	X	X	X	X	X

Note: Comparison 1: intervention group 1 versus control; comparison 2: intervention group 2 versus control; comparison 3: intervention group 1 versus intervention group 2

### Economic evaluation

The economic evaluation will focus on all three comparisons, i.e. intervention group 1 versus control group, intervention group 1 versus intervention group 2, and intervention group 2 versus control group.

The economic evaluation will be performed for all co-primary outcomes and QALYs. In the main analysis, the societal perspective will be applied, meaning that all costs will be included, irrespective of who pays or benefits from them. Unadjusted as well as adjusted cost differences across groups will be calculated for total and disaggregated costs. Ninety-five per cent CIs around those cost differences will be estimated using bias corrected and accelerated (BCA) bootstrapping, with 5000 replications. Cost and effect differences across groups will be estimated using seemingly unrelated regression (SUR) analyses, in which both are modelled simultaneously so that their possible correlation can be accounted for. Incremental cost-effectiveness ratios (ICERs) will be estimated by dividing the differences in costs by those in effects. The uncertainty surrounding the ICERs will be graphically illustrated by plotting bootstrapped cost-effect pairs on cost-effectiveness planes. Again, these bootstrapped cost-effect pairs will be estimated using the BCA bootstrap, with 5000 replications. An estimate of the joint uncertainty surrounding costs and effects will be provided by constructing cost-effectiveness acceptability curves (CEACs). These CEACs will provide an estimate of the probability of the interventions being cost-effective compared with each other. To assess the robustness of the results, three sensitivity analyses will be performed. In sensitivity analysis 1 (SA1), the healthcare perspective will be applied, meaning that only costs accruing to the formal Dutch healthcare system will be included in the analyses. In SA2, the human capital approach will be used instead of the friction cost approach for estimating absenteeism costs. In SA3, only data of patients with complete cost and effect measure values at all measurement points will be included.

## Discussion

During the last decade, there has been extensive debate about the effectiveness of epidural corticosteroids for treating sciatica. A 2020 meta-analysis, as part of the Dutch multidisciplinary guideline on sciatica (https://richtlijnendatabase.nl/richtlijn/lumbosacraal_radiculair_syndroom/lumbosacraal_radiculair_syndroom_-_startpagina.html) (and based on 6 systematic reviews [4, 5, 29–32]), showed a small, but statistically significant, short-term (< 3 months) effect for leg pain of epidural corticosteroids versus placebo (mean difference (MD), 0.94 on a 10 point visual analogue scale (VAS) [95% CI, 0.14 to 1.73]). Moreover, for physical functioning, a small not clinically relevant standardized mean difference of 0.32 (95% BI − 0.58 to 1.22) was found in favour of epidural steroids. However, the level of evidence of these studies according to the GRADE approach [33] was regarded as low. Therefore, this two-centre, randomized controlled, prospective, single-blind trial (STeroids Against Radiculopathy [STAR]) will provide valuable information about the effectiveness, cost-effectiveness and safety  of transforaminal epidural steroids in patients with sciatica shorter than 8 weeks, a subgroup that has hardly been addressed so far [34–36].

Unfortunately, however, we had to stop our trial prematurely, because of slow patient accrual, with only 53.4% of the required sample size being included in 2.5 years. Issues that affected slow patient accrual were the fact that (according to their guidelines) (https://richtlijnen.nhg.org/standaarden/lumbosacraal-radiculair-syndroom) Dutch general practitioners typically wait at least 6 weeks before referring patients with sciatica to a hospital; the fact that there were only 2 participating centres, and the fact that patients who believe in the superiority of epidural steroid injection over conservative treatment experience difficulty with being randomized and prefer active treatment with an epidural steroid injection. This is a well-known problem in back pain research [37, 38], but it will likely negatively affect the generalizability of our results to other (Dutch) sciatica patients and will result in the study being slightly underpowered for pain intensity and global perceived effect, but not for physical functioning.

## Conclusion

The STAR trial aims to provide evidence about TESIs in the treatment of acute sciatica (< 8 weeks). This statistical analysis plan details the study’s planned analyses, to aid transparency of results, and may assist the design of studies in the future.

### Trial status

Participant recruitment was completed in November 2019 and follow-up outcomes were collected till March 10, 2020. The data were available for analysis on May 15, 2020. The final analyses will commence after publication of the SAP.

## Supplementary Information

**Additional file 1.** Cost questionnaire.

## Data Availability

The datasets generated and/or analysed during the current study are not publicly available due to institution policy but are available from the corresponding author on reasonable request.

## Abbreviations

AE: Adverse event
AR: Adverse reaction
BCA: Bias corrected and accelerated
CEAC: Cost-effectiveness acceptability curve
CI: Confidence interval
CONSORT: Consolidated Standards of Reporting Trials
CRF: Case record form
EQ-5D-5 L: Euroqol-5 dimensions-5 levels
GCP: Good clinical practice
GPR: Global perceived recovery
GRADE: Grades of recommendation, assessment, development and evaluation
ICER: Incremental cost-effectiveness ratios
MCID: Minimal clinically important difference
MD: Mean difference
MICE: Multivariate Imputation by Chained Equations
MRC: Medical Research Council
MRI: Magnetic resonance imaging
NRS: Numerical rating scale
NTR: Nederlands Trial Register
PCQ: Productivity Cost Questionnaire
PRODISC: Productivity and Disease Questionnaire
QALY: Quality-adjusted life year
RCT: Randomized controlled trial
RDQ: Roland-Morris Disability Questionnaire
SA: Sensitivity analysis
SAP: Statistical analysis plan
SUR: Seemingly unrelated regression
TESI: Transforaminal epidural injections with steroids
TMF: Trial master file
VAS: Visual analogue scale
WHO: World Health Organization
WPQ: Work Performance Questionnaire
