# Supplementary material for: STeroids Against Radiculopathy (STAR) trial: a statistical analysis plan
Source: Trials. 2021 Jan 22;22:80. doi: 10.1186/s13063-020-05018-2 (PMC7821662; doi:10.1186/s13063-020-05018-2)
Supplement: Supplementary file 1 — Additional file 1. Cost questionnaire. [file 13063_2020_5018_MOESM1_ESM.docx]

**Supplemental file: cost questionnaire**
